# Co-occurring cyber and in-person victimisation of bullying and sexual harassment: the associations to depressive symptoms in Swedish adolescents

**DOI:** 10.1186/s12889-025-21989-w

**Published:** 2025-02-26

**Authors:** Albin Dahlström, Heléne Dahlqvist, Katja Gillander Gådin

**Affiliations:** Department of Health Sciences, Mid Sweden University, Sundsvall, 851 70 Sweden

**Keywords:** Mental health, Bullying, Sexual harassment, Adolescence, Depressive symptoms

## Abstract

**Background:**

Poor mental health has increased among adolescents in recent decades. Bullying and sexual harassment, both cyber and in-person, are each associated with increased depressive symptoms in adolescents and being victimised by co-occurring types is more common than just one single type of victimisation. The study aimed to investigate the association between co-occurring victimisation and depressive symptoms in adolescents when controlling for screen time, social support, physical activity, and personal relative affluence.

**Method:**

Cross-sectional survey data from 1211 respondents (50.1% girls) aged 15–16 were used to conduct modified Poisson regression with robust error variance analyses for girls and boys separately. Four scales were used to measure cyber and in-person bullying and sexual harassment, and CESD-R10 was used to measure depressive symptoms.

**Results:**

About 47% of girls and 20% of boys experienced all four types of victimisation, and about 12% of girls and 25% of boys experienced none of them. When controlled for all included variables, all number of victimisation types of bullying and/or sexual harassment were associated with depressive symptoms in girls. In comparison, only four types of victimisation were associated with depressive symptoms in boys.

**Conclusions:**

The study showed that co-occurring victimisation was associated with depressive symptoms even after controlling for other relevant factors in both genders. However, in girls, all numbers of victimisation were associated with depressive symptoms.

**Supplementary Information:**

The online version contains supplementary material available at 10.1186/s12889-025-21989-w.

## Background

Poor mental health is a complex concept, encompassing different conditions that cannot be attributed to a single specific risk factor. Instead, it results from a combination of various risk factors, each of which contributes to a certain likelihood of developing, for example, depression. Depression is related to social, psychological, and genetic risk factors [1]. Among adolescents, the prevalence of depressive symptoms has increased globally during recent decades, with adolescent girls consistently showing higher rates than boys [2]. These gender differences in depression and depressive symptoms emerge at least at the age of 12 and peak at the age of 13–15 for depression and at the age of 16 for depressive symptoms to decline and remain stable in adulthood [3]. The factors contributing to gender disparities in depressive disorders have yet to be clarified. While worse mental health in girls is globally widespread, the gender gap in mental health outcomes seems to be more prominent in more affluent and more gender-equal countries [4]. This might be because the female roles in more gender-equal countries are less pronounced, with potential conflict between roles and balancing norms of femininity with potential contradictive norms of gender equality, which is particularly stressful for adolescent girls [5]. Other contributing explanations could be that when girls go through puberty, they encounter more peer sexual harassment than boys [6, 7]. In Sweden, the increase in symptoms of anxiety, depression and somatic symptoms has been more pronounced among girls than boys [8].

Depression in adolescents is associated with various factors, including genetic predispositions (e.g., family history of depression), personality traits (e.g., neuroticism), and social stressors such as early life trauma, stressful life events, disrupted caregiving, or bullying [1]. Common risk factors for depression among adolescents include low socioeconomic status (SES) [9, 10], as well as the time spent on social media, its intensity of use and problematic usage patterns [11]. Longitudinal findings suggest that the link between time spent on social media and mental health weakens when accounting for inadequate sleep, lower physical activity and cyberbullying, especially in girls [12]. Research on the link between gaming and depressive symptoms in adolescents presents mixed findings, suggesting a bi-directional relationship. Studies focusing on gaming addiction or problematic gaming behaviour, rather than merely measuring time spent, are more likely to reveal a significant association [13]. Sedentary behaviours have been associated with depression, and physical activity is positively associated with mental well-being [14]. However, Biddle et al. [15] suggest that there is no evidence for a dose-response relationship in adolescents.

Social support from parents, family, and teachers has consistently been shown to protect against depression. However, evidence regarding the protective effect of support from friends and general perceived support is less consistent [9, 16].

Peer victimisation during adolescence, which includes bullying and sexual harassment [17], is a significant public health issue with far-reaching consequences. While the prevalence of peer victimisation differs across cultures, samples, measurements and research methods, it remains widespread [18, 19]. Bullying and sexual harassment can be divided into cyber and in-person, meaning there are four types of this sort of victimisation [20]. Victimised adolescents are at increased risk for mental health issues, such as anxiety, depression, low self-esteem and suicidal ideation [21–25]. These psychological effects often persist into adulthood, contributing to long-term emotional and social difficulties [26]. Additionally, victims may experience physical health problems, including headaches, sleep disturbances, and other stress-related conditions, as a result of the chronic stress and emotional distress caused by victimisation [23]. Previous studies have shown that bullying has a stronger effect on the risk for depression among girls than boys [27, 28], and girls are more adversely affected by sexual harassment [22, 29, 30]. The negative social impact of nonconsensual sharing of intimate images (which is part of cyber sexual harassment) is worse among girls than boys and can lead to even more sexual harassment, bullying, and victim-blaming [24, 31].

Few studies have examined the co-occurrence of cyber and in-person bullying and sexual harassment victimisation. A previous Swedish study [20] found that adolescents were more likely to experience a combination of cyber and in-person bullying and sexual harassment than any single type of victimisation.

### The current study

Despite extensive research on bullying and sexual harassment as independent contributors to adolescent depression, the co-occurrence of these victimisation types- both cyber and in-person -remains underexplored. Additionally, the association between co-occurring victimisation and depressive symptoms, while controlling for other risk and protective factors such as SES, social support, and physical activity, requires further investigation.

### Aim of the study

The aim of this study was to investigate the association between co-occurring bullying and sexual harassment victimisation and depressive symptoms in adolescent girls and boys while controlling for physical activity, social media and gaming, support from family, teachers, and friends, and personal relative affluence.

### Research questions

Does the likelihood of depressive symptoms increase with the number of victimisation forms?

Are there gender differences in the associations between the number of victimisation forms and depressive symptoms?

## Method

### Context

The current study was conducted during the spring of 2023 in a northern county of Sweden with 243 265 inhabitants (population density 11.3/km²), distributed in all seven municipalities, ranging from a medium-sized city of 99 361 inhabitants to the smallest municipality with a population of 9143 [32]. In Sweden, compulsory schooling starts at age six and reaches grade 9 (age 15–16).

### Study population and procedure

A cross-sectional design was employed to collect data through a web-based survey conducted from January to April 2023. The web survey was comprehensive and covered the following topics (in order): background information (including SES), health issues, online sexual harassment, online bullying, time spent on social media, gaming, in-person sexual harassment, in-person bullying, coping strategies for victimisation, intimate partner violence, honour-related oppression, social support, and school related issues.

The study targeted 9th-grade pupils aged 15–16 during the school year 2022/2023. Out of 41 eligible schools in the county comprising 2798 pupils in 9th grade, 24 schools were randomly selected, involving 1502 pupils representing both public and independent schools. All schools in the county were initially provided with general information about the study and that they would undergo randomisation. Following randomisation on all eligible schools in the county, a more detailed briefing was conducted with the selected schools, seeking the schools’ consent for participation.

An information letter was distributed to pupils to ensure their informed consent regarding participation in the web survey. The survey ensured anonymity and was sent via a link to the pupils’ school, facilitated by the school through a teaching platform. It was administered to the pupils during class. Pupil inclusion criteria comprised pupils in 9th grade, aged at least 15, and able to respond to the survey independently.

Except in three schools, where only school staff were present, the teacher and a research team member were present to monitor and answer any questions. The team members provided comprehensive oral information about the study and ethical considerations to ensure that all potential respondents were well-informed. The questionnaire was designed only to allow respondents to enter it after confirming that they had received the necessary ethical information and were participating voluntarily. The pupils were seated on separate desks in the classroom, and desktop screens were used to ensure privacy. Pupils who finished the questionnaire early were required to remain in class and do schoolwork. The pupils received information at the end of the questionnaire that they could turn to school healthcare after the survey if needed. The study was conducted in accordance with the Declaration of Helsinki and approved by the Swedish Ethical Review Authority with issue number Dnr 2022-05776-01. A total of 1232 pupils responded, giving a response rate of 82%. Following the data quality check, 1211 respondents (50.1% girls and 49.9% boys) were included in the analyses, corresponding to a response rate of 81%. Twenty-one respondents were excluded due to either not completing the questionnaire or providing random or facetious answers.

### Measures

The questionnaire was first tested on adolescents aged 13–18 to test face validity, text quality, and adaption to the target group. It covered various areas, including background information, health, experiences of cyber- and in-person bullying, and sexual harassment. Based on the abovementioned studies, physical activity, personal relative affluence, family, friends, and teacher support, as well as time spent on social media and gaming, were chosen as control variables. The control variables were tested using Spearman’s correlation to check that there were no high correlations causing multicollinearity in the analyses.

### Depressive symptoms

Depressive symptoms were measured using the Center for Epidemiologic Studies Depression Scale Revised 10 (CESD-R10), a revised version of the CESD-20 [33] consisting of 10 items. Participants answered a four-graded scale (ranging from “rarely or never” to “all of the time”). The coding adhered to instructions, setting a cut-off at 10 points or more out of 30 points to indicate the presence of depressive symptoms. Personal mean imputation was used for cases with ≤ 20% missing items. The validity and reliability of the instrument on adolescents aged 15–21 have been proven previously [34]. Cronbach’s alpha was 0.864 in the current sample.

### Cyber and in-person bullying and sexual harassment

Cyber and in-person bullying and sexual harassment (SH) were assessed using four distinct constructs, referred to as “victimisation types”; see items for all four victimisation types in Appendix 1. The respondents reported their experiences in the last six months on a four-graded scale ranging from “never” to “many times”. Having at least one experience once or more was accounted as being victimised. Previous studies have used this definition [20, 22]. Depending on how many types of victimisations the pupils reported, they were divided into five groups: 0–4 types, where 0 meant not victimised by any of the types, and four meant victimised by all four types: cyberbullying, cyber sexual harassment, in-person bullying and in-person sexual harassment.

In-person bullying was evaluated using a modified version of the California Bullying Victimization Scale (CBVS). Instead of seven items, six items were used, excluding the item about sexualised offences as it was captured by the in-person sexual harassment (SH) instrument. The scale has previously demonstrated good internal consistency, test-retest reliability, and concurrent and predictive validity on adolescents [35]. Cronbach’s alpha was 0.819 in the current sample.

Cyberbullying was measured by six items derived from the CBVS scale [35] but adapted to a digital context and from Wong and McBride [36]. Cronbach’s alpha was 0.835 in the current sample.

In-person SH was assessed using 12 items derived from the American Association of University Women (AAUW) [37] and Gruber and Fineran [38] and adapted to a contemporary context. Cronbach’s alpha was 0.880 in the current sample.

Cyber SH was measured by 14 items derived from a literature review [19, 39–41]. Cronbach’s alpha was 0.920 in the current sample.

### Physical activity

Physical activity was measured through a question from The Public Health Agency of Sweden [42] by asking respondents to indicate how often they engage in physical activities in their leisure time (outside of school hours) to the point of getting short of breath or sweating. The response alternatives were every day, almost every day, some days a week, once a week, one to two times a month, less than once a month, and never. Once a week or less was regarded as a low level of physical activity and coded as 1.

### Social support

Family, friends, and teacher support was measured by four questions each from Health Behaviour in School-aged Children (HBSC) [43]. The five response alternatives ranged from “always”, “often”, “sometimes”, and “rarely” to “never” and were coded as 0 (“never”) to 4 (“always”), giving a maximum of 16 points per scale. The whole sample was divided into tertiles. The lowest tertial was considered as low support, coded as 1, with the cut-off scores < 12 for family support, < 11 for friend support and < 9 for teacher support. Spearman’s correlation test was used to check that there was no multicollinearity between the types of support.

### Social media and gaming

Social media and gaming habits were assessed separately by asking about how many hours were spent on each activity per day − weekdays and weekends. The response options were: “not engaging in it at all”, “not every day”, “less than 2 hours”, “2–4 hours”, “4–7 hours”, and “more than 7 hours”. The response alternatives were divided into ≤ 4 h a day and > 4 h a day. Weekdays and weekends were merged for both social media and gaming. The risk value (coded 1) was set to > 4 h a day.

### Personal relative affluence

The concept of personal relative affluence was used to measure the pupils’ economic position. It was measured by the question: “If you think about the past six months. How often has it happened that you could not afford to buy and/or do the same things as your friends?” Response options were: “always”, “often”, “sometimes”, “rarely”, and “never”. Pupils indicating “always”, “often” or “sometimes” were scored as 1, being unable to buy/do the same as friends, labelled low affluence. The question has been used in previous studies as a proxy for adolescents’ economic situation [22, 44].

### Data analysis

All variables were first analysed for potential gender differences using a chi-square test with a significance level of < 0.05. Because most variables showed gender differences, each gender was analysed separately. The current study applied modified Poisson regression with a robust error variance method to enhance the precision of the estimates [45]. This method is often favoured for deriving direct relative risk (RR) estimates, making it particularly suitable for assessing population-level risk [46]. All variables were tested to attain the unadjusted RR and test their association with depressive symptoms.

The last step was to examine the relationship between experiencing 1–4 types of victimisation and depressive symptoms, together with the control variables: gaming and social media, physical activity, support from family, friends, and teachers, and personal relative affluence. SPSS 29 was used to conduct the statistical analysis.

## Result

About one in 10 girls and one in four boys were not victimised by any of the four types, compared with almost half of the girls and one in five boys who reported all four types of victimisation. Reporting of depressive symptoms was far more common among girls than boys, 61% and 26.4%, respectively (Table 1).

**Table 1:** Prevalence in each variable for girls and boys

Variable	Girls % (n) (*n* = 607)	Boys % (n) (*n* = 604)	*P*-value (95%)
**Outcome variable**			
Depressive symptoms Yes	61.0 (362)	26.4 (152)	< 0.001
**Predictors**			
**Number of victimisation types experienced**			
0	11.6 (69)	25.4 (151)	
1	10.3 (61)	20.0 (119)	
2	11.8 (70)	16.0 (95)	< 0.001
3	19.9 (118)	18.5 (110)	
4	46.5 (276)	20.0 (119)	
**Personal relative affluence**			
Low	19.8 (119)	18.4 (111)	0.539
**Physical activity**			
Low	38.1 (227)	26.2 (155)	< 0.001
**Social media**			
> 4 h/day	52.8 (312)	23.3 (136)	< 0.001
**Gaming**			
> 4 h/day	3.4 (20)	21.6 (126)	< 0.001
**Family support**			
Low	42.5 (248)	24.8 (138)	< 0.001
**Friend support**			
Low	33.4 (194)	34.6 (190)	0.683
**Teacher support**			
Low	45.9 (266)	35.8 (194)	< 0.001

As shown in Table 2, there was a significantly higher prevalence of cyber and in-person bullying and SH among girls compared to boys. Cyber SH and in-person bullying were the most common victimisation types in both genders.

**Table 2:** Prevalence of each victimisation type and unadjusted association with depressive symptoms among girls and boys

Victimisation type	Prevalence % (*n*)	Unadjusted OR (95% CI)	*P*-value (95%)
Girls			
Cyberbullying	66 (385)	4.10 (2.85–5.91)	< 0.001
Cyber SH	75 (443)	4.95 (3.32–7.37)	< 0.001
In-person bullying	76 (445)	4.81 (3.20–7.25)	< 0.001
In-person SH	65 (386)	5.17 (3.58–7.47)	< 0.001
Boys			
Cyberbullying	45 (257)	2.87 (1.93–4.26)	< 0.001
Cyber SH	51 (299)	3.01 (2.01–4.50)	< 0.001
In-person bullying	53 (299)	2.06 (1.38–3.08)	< 0.001
In-person SH	45 (260)	2.54 (1.72–3.76)	< 0.001

The result presented in Table 3 shows that the unadjusted RR for all included variables were significantly associated with depressive symptoms, except gaming > 4 h a day for girls. When adjusting for the control variables, the analysis revealed that all number of victimisation types remained significantly associated with depressive symptoms. In the adjusted model, girls who reported low levels of physical activity (95% CI: 1.05–1.31), low family support (95% CI: 1.26–1.63), low friend support (95% CI: 1.01–1.25), and low personal relative affluence (95% CI: 1.10–1.36) had significantly higher estimated RR for depressive symptoms compared to reference groups. Spending > 4 h a day on social media or gaming and low support from teachers did not show a significant association with depressive symptoms in this adjusted model for girls.

**Table 3:** Associations between predictors and depressive symptoms in girls

Predictor		Girls with depressive symptoms % (*n*)	Unadjusted		Adjusted		
			RR	95% CI for RR		RR	95% CI for RR
Number of victimisation types experienced	0	17.6 (12)	1			1	
	1	41.7 (25)	2.73**	1.43–5.21		2.43**	1.30–4.56
	2	41.2 (28)	2.46**	1.29–4.70		2.13*	1.14–3.97
	3	63.2 (74)	4.06***	2.26–7.30		3.38***	1.91–5.98
	4	79.7 (220)	5.10***	2.88–9.04		3.73***	2.12–6.56
Social media	≤ 4/h/day	52.9 (145)	1			1	
	> 4 h/day	68.6 (214)	1.29***	1.12–1.47		1.06	0.94–1.19
Gaming	≤ 4/h/day	60.8 (345)	1			1	
	> 4 h/day	70.0 (14)	1.22	0.92–1.61		0.97	0.71–1.33
Physical activity level	High	55.2 (201)	1			1	
	Low	69.9 (158)	1.28***	1.13–1.46		1.17**	1.05–1.31
Family support	Higher	44.6 (148)	1			1	
	Low	83.8 (207)	1.90***	1.66–2.17		1.43***	1.26–1.63
Friend support	Higher	53.0 (203)	1			1	
	Low	77.7 (150)	1.46***	1.29–1.64		1.12*	1.01–1.25
Teacher support	Higher	48.9 (152)	1			1	
	Low	75.8 (201)	1.55***	1.36–1.77		1.11	0.98–1.25
Personal relative affluence	High	55.5 (263)	1			1	
	Low	82.8 (96)	1.48***	1.32–1.67		1.22***	1.10–1.36

RR Relative Risk

* *p* < 0.05, **p < 0.01, ***p < 0.001

As shown in Table 4, for boys, all variables in the unadjusted model were associated with depressive symptoms, except reporting one to two victimisation types. In the adjusted model, only victimisation by four types remained associated with depressive symptoms. Among the control variables, compared with the reference groups, significantly higher RR for depressive symptoms was shown for boys reporting gaming > 4 h a day (95% CI: 1.03–1.80), having low level of physical activity (95% CI: 1.31–2.25), low family support (95% CI: 1.40–2.64), low level of teacher support (95% CI: 1.00-1.84) and low personal relative affluence (95% CI: 1.09–1.96). In the adjusted model for boys, neither spending > 4 h on social media a day nor low support from friends was found to be associated with depressive symptoms.

**Table 4:** Associations between predictors and depressive symptoms in boys

Predictor		Boys with depressive symptoms % (*n*)	Unadjusted		Adjusted		
			RR	95% CI for RR		RR	95% CI for RR
Number of victimisation types experienced	0	13.2 (19)	1			1	
	1	20.4 (23)	1.35	0.75–2.43		1.12	0.65–1.94
	2	22 (20)	1.54	0.85–2.81		1.19	0.68–2.06
	3	27.1 (29)	1.97*	1.16–3.37		1.51	0.89–2.55
	4	49.6 (57)	3.45***	2.16–5.53		1.91**	1.19–3.07
Social media	≤ 4/h/day	23.1 (101)	1			1	
	> 4 h/day	35.7 (43)	1.54**	1.14–2.09		1.15	0.87–1.51
Gaming	≤ 4/h/day	23.0 (101)	1			1	
	> 4 h/day	36.1 (44)	1.56**	1.16–2.11		1.36*	1.03–1.80
Physical activity level	High	21.9 (92)	1			1	
	Low	39.2 (58)	1.93***	1.46–2.57		1.72***	1.31–2.25
Family support	Higher	17.7 (72)	1			1	
	Low	51.2 (66)	2.93***	2.23–3.84		1.92***	1.40–2.64
Friend support	Higher	19.6 (68)	1			1	
	Low	38.3 (70)	2.07***	1.55–2.75		1.27	0.94–1.72
Teacher support	Higher	37.7 (69)	1			1	
	Low	19.9 (68)	1.91***	1.44–2.55		1.36*	1.00-1.84
Personal relative affluence	High	22.6 (106)	1			1	
	Low	43.8 (46)	2.01***	1.50–2.69		1.46*	1.09–1.96

RR Relative Risk

* *p* < 0.05, **p < 0.01, ***p < 0.001

## Discussion

The current study set out to examine co-occurring bullying and sexual harassment victimisation association with depressive symptoms in adolescents. Regardless of the number of victimisation types, the association with depressive symptoms persisted for girls even after controlling for physical activity, social media and gaming time, support from family, teachers, and friends, as well as personal relative affluence. The likelihood of depressive symptoms increased with the number of victimisation types, except for being victimised by one type, which showed a slightly higher relative risk than two types among girls. However, among boys, only reporting victimisation of all four types of bullying and sexual harassment (SH) remained associated with depressive symptoms in the adjusted model.

The findings indicate that all four types of victimisation examined are necessary for a significant increase in the risk ratio (RR) of depressive symptoms among boys. This finding might seem counterintuitive, as one would expect the dose-response effect to be similar for both girls and boys. Gender differences in the association between cyber and in-person bullying, SH victimisation, and depressive symptoms may be explained by previous research findings. Sexual harassment has been associated with later depressive symptoms among adolescent girls but not among boys in a longitudinal study [47]. Anhedonia, a key dimension of depressive symptoms, was found to precede SH in both genders. Among boys, depressive symptoms specifically preceded SH in the form of sexual name-calling, suggesting that depressive symptoms may increase boys’ vulnerability to this type of victimisation. For girls, sexual harassment victimisation (including physical harassment, name-calling, and public display) remained stable over a two-year period, with repeated experiences. However, this pattern was less evident for boys, except in cases of sexual name-calling [47]. Bullying is a predictor of depressive symptoms among both girls and boys but has been shown to have a stronger effect on the risk for depression among girls than boys [27, 28], which contributes to explaining our findings. Boys are more often involved as bullies than girls [48, 49], and bullies, in general, compared to non-bullies, have been shown to be more aggressive and have more sexist attitudes against women, male homosexuality and gender-nonconforming people [49]. The same could be said about SH, with boys more involved as perpetrators than girls [50, 51], and bullying can be one predictor of later SH perpetration among adolescents [52]. Girls also seem to be more adversely affected by SH than boys [22, 38]. Gruber and Fineran [38] found that SH had a more harmful impact than bullying for both sexes. Sexual harassment is a distinctly different experience compared to bullying, and the sexual content is critical. It is linked to the view of gender and sexuality and that certain groups are subordinated (females and sexual minorities) [38]. Victims of SH have reported experiencing more trauma than those who were victimised in non-sexual ways [31, 53]. Sexualised victimisation probably means something different for girls than for boys concerning depressive symptoms. Previous research has demonstrated that sexual double standards create worse social and sexual stigma, shaming and victim blaming for girls [31, 53]. One study exemplifies that while boys could shrug off the non-consensual sharing of their nudes with laughter, girls faced sexual shame, stigma and blame for their victimisation [31]. Both bullying and SH could excessively victimise girls and minority groups because of the experience of a social power imbalance where girls are more subjected to hegemonic masculinity [38].

In the current study, cyberbullying and in-person bullying were clearly distinguished from sexualised victimisation. However, some bullying behaviours may be closely related to one’s sexuality. Within bullying, “appearance-based” bullying may harm everyone, but because of stronger norms concerning appearance, it may strike harder against girls. Both weight-based and SH have shown associations with depressive symptoms among girls and lower self-esteem among both boys and girls [54]. The greater adverse impacts on the risk of depressive symptoms among girls because of bullying and SH victimisation may explain the stronger associations for co-occurring victimisations among girls in the current study.

In line with previous studies [9, 14, 16], girls and boys shared most predictors associated with higher RR for depressive symptoms, such as low level of physical activity, low support from family and low personal relative affluence. Low support from friends was significantly associated with depressive symptoms in girls but not in boys. Boys reporting low support from teachers, as well as much time spent on gaming, had a higher risk of depressive symptoms. Rueger et al. [55] found that family support and general peer support were the strongest sources of support, protecting against depression, followed by support from teachers, which was stronger than support from close friends [55]. The current study’s result is inconsistent with Rueger et al. [55], who could not find any gender differences in the association between social support and depression among adolescents.

Time spent on gaming and depressive symptoms are predictors of internet gaming disorder (IGD). IGD, which is more common among adolescent boys and more likely to develop in boys compared to girls [56], has been shown to predict depressive symptoms [57]. This could explain the observed association between time spent on gaming and depressive symptoms in boys in the current study. Motivation, content and interaction in social media and gaming probably play a significant role in depressive symptoms, not only the time spent.

### Methodological considerations

A limitation of this study is the cross-sectional design, which cannot clarify the direction of the associations. Something that could limit the understanding of the results is that the severity and frequency of the acts are not displayed. Also, self-reported data can be influenced by social desirability bias and memory errors [58]. At the same time, it is practically and ethically challenging to obtain these data using any other method. The research team took many measurements to ensure the anonymity and privacy of the pupils when they responded to the survey. Considering the nature of some questions, the pupils were informed that they could turn to the school healthcare or other support.

The current study used the modified Poisson regression that includes the robust error variance procedure to analyse the association between victimisation and depressive symptoms. The modified Poisson regression procedure is considered highly reliable regarding both relative bias and the percentage of confidence interval coverage [45]. Relative risk is often preferred over odds ratios because logistic regression analyses tend to overestimate odds ratios. In this context, modified Poisson regression emerges as a better alternative for analysing cross-sectional studies with binary outcomes than logistic regression [59].

The study’s strengths were the randomisation of schools and the high response rate. The presence of research team members contributed to this, as well as the fact that the schools prioritised the topic. The relatively large sample enabled adjustment for the control variables. Pilot testing of the questions was conducted to check face validity.

### Implications for future research

Future research should further investigate the complex interplay of cyber and in-person bullying and sexual harassment victimisation and their cumulative effects on mental health over time. There are also other possible outcomes of co-occurring victimisation that should be studied, such as anxiety, self-harm and subjective health complaints. The significant gender differences in victimisation and their associations with depressive symptoms suggest the need for gender-specific analyses in future studies. Understanding these differences can aid in developing more effective prevention and support strategies. This must also be regarded when working preventively in schools and other arenas for adolescents. Identifying additional protective factors and their potential to mitigate the negative impacts of victimisation could inform the development of comprehensive intervention programs.

## Conclusion

Most girls reported three to four types of co-occurring bullying and sexual harassment victimisation, and the associations with depressive symptoms remained strong after controlling for other predictors. For boys, only experiencing all four types of victimisation remained associated with depressive symptoms. A higher level of physical activity, support from family, friends and teachers, and higher personal relative affluence had independent associations. Still, they did not remove the association between co-occurring victimisation and depressive symptoms.

This study emphasises the importance of considering the co-occurring victimisation of cyber and in-person bullying and sexual harassment and underlying gender differences when studying adolescents’ mental health. Promoting physical activity and social support can benefit adolescents’ mental health, but addressing co-occurring victimisation is crucial for interventions meaningful for adolescents’ everyday lives.

## Electronic supplementary material

Below is the link to the electronic supplementary material.

Supplementary Material 1

## Data Availability

The corresponding author will share the article’s data on reasonable request.

## Abbreviations

AAUW: American Association of University Women
CBVS: California Bullying Victimization Scale
CESD: Center for Epidemiologic Studies Depression
IGD: Internet gaming disorder
SH: Sexual harassment

## References

[1] Thapar A, Eyre O, Patel V, Brent D. Depression in young people. Lancet. 2022;400:617–31. 35940184 10.1016/S0140-6736(22)01012-1

[2] Shorey S, Ng ED, Wong CHJ. Global prevalence of depression and elevated depressive symptoms among adolescents: A systematic review and meta-analysis. Br J Clin Psychol. 2022;61:287–305. 34569066 10.1111/bjc.12333

[3] Salk RH, Hyde JS, Abramson LY. Gender differences in depression in representative National samples: Meta-analyses of diagnoses and symptoms. Psychol Bull. 2017;143:783–822. 28447828 10.1037/bul0000102 PMC5532074

[4] Campbell OLK, Bann D, Patalay P. The gender gap in adolescent mental health: A cross-national investigation of 566,829 adolescents across 73 countries. SSM Popul Health. 2021;13:100742. 33748389 10.1016/j.ssmph.2021.100742 PMC7960541

[5] Hopcroft RL, Bradley DB. The sex difference in depression across 29 countries. Soc Forces. 2007;85:1483–507.

[6] Lindberg SM, Grabe S, Hyde JS, Gender. Pubertal development, and peer sexual harassment predict objectified body consciousness in early adolescence. J Res Adolesc. 2007;17:723–42.

[7] Skoog T, Bayram Özdemir S. Explaining why early-Maturing girls are more exposed to sexual harassment in early adolescence. J Early Adolesc. 2016;36:490–509.

[8] Blomqvist I, Henje Blom E, Hägglöf B, Hammarström A. Increase of internalized mental health symptoms among adolescents during the last three decades. Eur J Public Health. 2019;29:925–31. 30859217 10.1093/eurpub/ckz028 PMC6761841

[9] Shore L, Toumbourou JW, Lewis AJ, Kremer P, Review. Longitudinal trajectories of child and adolescent depressive symptoms and their predictors– a systematic review and meta-analysis. Child Adolesc Ment Health. 2018;23:107–20. 32677332 10.1111/camh.12220

[10] Reiss F. Socioeconomic inequalities and mental health problems in children and adolescents: A systematic review. Soc Sci Med. 2013;90:24–31. 23746605 10.1016/j.socscimed.2013.04.026

[11] Cunningham S, Hudson CC, Harkness K. Social media and depression symptoms: a Meta-Analysis. Res Child Adolesc Psychopathol. 2021;49:241–53. 33404948 10.1007/s10802-020-00715-7

[12] Viner RM, Gireesh A, Stiglic N, Hudson LD, Goddings A-L, Ward JL, Nicholls DE. Roles of cyberbullying, sleep, and physical activity in mediating the effects of social media use on mental health and wellbeing among young people in England: a secondary analysis of longitudinal data. Lancet Child Adolesc Health. 2019;3:685–96. 31420213 10.1016/S2352-4642(19)30186-5

[13] Shin M, Juventin M, Wai Chu JT, Manor Y, Kemps E. Online media consumption and depression in young people: A systematic review and meta-analysis. Comput Hum Behav. 2022;128:107129.

[14] Rodriguez-Ayllon M, Cadenas-Sánchez C, Estévez-López F, Muñoz NE, Mora-Gonzalez J, Migueles JH, et al. Role of physical activity and sedentary behavior in the mental health of preschoolers, children and adolescents: A systematic review and meta-analysis. Sports Med. 2019;49:1383–410. 30993594 10.1007/s40279-019-01099-5

[15] Biddle SJH, Ciaccioni S, Thomas G, Vergeer I. Physical activity and mental health in children and adolescents: an updated review of reviews and an analysis of causality. Psychol Sport Exerc. 2019;42:146–55.

[16] Gariépy G, Honkaniemi H, Quesnel-Vallée A. Social support and protection from depression: systematic review of current findings in Western countries. Br J Psychiatry. 2016;209(4):284–93. 27445355 10.1192/bjp.bp.115.169094

[17] Crowley BZ, Cornell D. Associations of bullying and sexual harassment with student well-being indicators. Psychol Violence. 2020;10:615–25.

[18] Biswas T, Scott JG, Munir K, Thomas HJ, Huda MM, Hasan MM, et al. Global variation in the prevalence of bullying victimisation amongst adolescents: role of peer and parental supports. EClinicalMedicine. 2020;20:100276. 32300737 10.1016/j.eclinm.2020.100276 PMC7152826

[19] Reed E, Wong A, Raj A. Cyber sexual harassment: A summary of current measures and implications for future research. Violence against Women. 2020;26:1727–40. 31631815 10.1177/1077801219880959

[20] Dahlqvist H, Svensson Å, Gillander Gådin K. Co-occurrence of online and offline bullying and sexual harassment among youth in Sweden: implications for studies on victimization and health a short communication. Int J Circumpolar Health. 2022;81. 10.1080/22423982.2022.2130362 PMC9543120 36178257

[21] Edwards L, Kontostathis AE, Fisher C. Cyberbullying, race/ethnicity and mental health outcomes: A review of the literature. Media Commun. 2016;4:71–8.

[22] Dahlqvist HZ, Gillander Gådin K. Online sexual victimization in youth: predictors and cross-sectional associations with depressive symptoms. Eur J Public Health. 2018;28:1018–23. 29868848 10.1093/eurpub/cky102

[23] Moore SE, Norman RE, Suetani S, Thomas HJ, Sly PD, Scott JG. Consequences of bullying victimization in childhood and adolescence: A systematic review and meta-analysis. World J Psychiatry. 2017;7:60. 28401049 10.5498/wjp.v7.i1.60 PMC5371173

[24] Schmidt F, Varese F, Larkin A, Bucci S. The mental health and social implications of nonconsensual sharing of intimate images on youth: A systematic review. Trauma Violence Abuse. 2024;25:2158–72. 37970838 10.1177/15248380231207896 PMC11155207

[25] Tran HGN, Thai TT, Dang NTT, Vo DK, Duong MHT. Cyber-Victimization and its effect on depression in adolescents: A systematic review and Meta-Analysis. Trauma Violence Abuse. 2023;24:1124–39. 34689637 10.1177/15248380211050597

[26] Takizawa R, Maughan B, Arseneault L. Adult health outcomes of childhood bullying victimization: evidence from a Five-Decade longitudinal British birth cohort. Am J Psychiatry. 2014;171:777–84. 24743774 10.1176/appi.ajp.2014.13101401

[27] Tong Y, Xie F, Wen X, Li Y, Yuan M, Zhang X et al. Longitudinal association between bullying victimization and depressive symptoms in Chinese early adolescents: the effect of life satisfaction. Depress Anxiety. 2024;2024(1). 10.1155/2024/6671415 PMC11918514 40226671

[28] Song Q, Yuan T, Hu Y, Liu X, Fei J, Zhao X, et al. The effect of peer victimization during adolescence on depression and gender differences: A systematic review and Meta-analysis. Trauma Violence Abuse. 2024;25:2862–76. 38347760 10.1177/15248380241227538

[29] Gruber J, Fineran S. Sexual harassment, bullying, and school outcomes for high school girls and boys. Violence against Women. 2016;22:112–33. 26270385 10.1177/1077801215599079

[30] Copp JE, Mumford EA, Taylor BG. Online sexual harassment and cyberbullying in a nationally representative sample of teens: prevalence, predictors, and consequences. J Adolesc. 2021;93:202–11. 34801812 10.1016/j.adolescence.2021.10.003

[31] Ringrose J, Regehr K. Recognizing and addressing how gender shapes young People’s experiences of image-based sexual harassment and abuse in educational settings. J Soc Issues. 2023;79:1251–81.

[32] Statistikmyndigheten [Statistics Sweden]. Befolkningsstatistik [Population statistics]. Available at: https://www.scb.se/hitta-statistik/statistik-efter-amne/befolkning/befolkningens-sammansattning/befolkningsstatistik/ (4 July 2024, date last accessed).

[33] Radloff LS. The CES-D scale. Appl Psych Meas. 1977;1(3):385–401.

[34] Bradley KL, Bagnell AL, Brannen CL. Factorial validity of the center for epidemiological studies depression 10 in adolescents. Issues Ment Health Nurs. 2010;31:408–12. 20450343 10.3109/01612840903484105

[35] Atik G, Guneri OY. California bullying victimization scale: validity and reliability evidence for the Turkish middle school children. Procd Soc Behv. 2012;46:1237–41.

[36] Wong N, McBride C. Fun over conscience: Fun-seeking tendencies in cyberbullying perpetration. Comput Hum Behav. 2018;86:319–29.

[37] AAUW. Hostile hallways: bullying, teasing, and sexual harassment in school. Am J Health Educ. 2001;32:307–9.

[38] Gruber JE, Fineran S. Comparing the impact of bullying and sexual harassment victimization on the mental and physical health of adolescents. Sex Roles. 2008;59:1–13.

[39] Powell A, Henry N. Technology-Facilitated sexual violence victimization: results from an online survey of Australian adults. J Interpers Violence. 2019;34:3637–65. 27697966 10.1177/0886260516672055

[40] Montiel I, Carbonell E, Pereda N. Multiple online victimization of Spanish adolescents: results from a community sample. Child Abuse Negl. 2016;52:123–34. 26724825 10.1016/j.chiabu.2015.12.005

[41] Walters GD, Espelage DL. Assessing the relationship between cyber and traditional forms of bullying and sexual harassment: stepping stones or displacement? Cyberpsychology (Brno) 2020;14.

[42] Folkhälsomyndigheten [The Public Health Agency of Sweden]. Skolbarns hälsovanor i Sverige 2021/22. Nationella resultat. [Health behaviour in school-aged children in Sweden 2021/22. National results]. Available at: https://www.folkhalsomyndigheten.se/publikationer-och-material/publikationsarkiv/s/skolbarns-halsovanor-i-sverige-2021-2022-nationella-resultat/ (4 July 2024, date last accessed).

[43] WHO E. Spotlight on adolescent health and well-being. Findings from the 2017/2018 Health Behaviour in School-aged Children (HBSC) survey in Europe and Canada. International report. Volume 1. Key findings. Copenhagen: World Health Organization. Regional Office for Europe. 2020. Available at: https://iris.who.int/handle/10665/332091 (4 July 2024, date last accessed).

[44] Zetterström Dahlqvist H, Landstedt E, Gådin KG. Depressive symptoms and the associations with individual, psychosocial, and structural determinants in Swedish adolescents. Health. 2012;04:881–9.

[45] Zou G. A modified Poisson regression approach to prospective studies with binary data. Am J Epidemiol. 2004;159:702–6. 15033648 10.1093/aje/kwh090

[46] Jean-Louis G, Turner AD, Seixas A, Jin P, Rosenthal DM, Liu M, Avirappattu G. Epidemiologic methods to estimate insufficient sleep in the US population. Int J Environ Res Public Health. 2020;17:9337. 33327388 10.3390/ijerph17249337 PMC7764851

[47] Dahlqvist HZ, Landstedt E, Young R, Gådin KG. Dimensions of peer sexual harassment victimization and depressive symptoms in adolescence: A longitudinal Cross-Lagged study in a Swedish sample. J Youth Adolesc. 2016;45:858–73. 26910524 10.1007/s10964-016-0446-x PMC4826406

[48] Baldry AC, Farrington DP, Sorrentino A. School bullying and cyberbullying among boys and girls: roles and overlap. J Aggress Maltreatment Trauma. 2017;26:937–51.

[49] Carrera-Fernández MV, Cid-Fernández XM, Almeida A, González-Fernández A, Lameiras-Fernández M. Me and Us versus the others: troubling the bullying phenomenon. Youth Soc. 2021;53:417–38.

[50] Clear ER, Coker AL, Cook-Craig PG, Bush HM, Garcia LS, Williams CM, et al. Sexual harassment victimization and perpetration among high school students. Violence against Women¨. 2014;20:1203–19. 25288593 10.1177/1077801214551287

[51] Espelage DL, Liu GS, Valido A, Kuehl T, Basile KC, Nickodem KK. Violence perpetration prevalence among Colorado (United States) high school students across gender, Racial/ethnic, and sexual identities. Prev Med. 2022;161:107146. 35810935 10.1016/j.ypmed.2022.107146 PMC9733587

[52] Espelage DL, Basile KC, De La Rue L, Hamburger ME. Longitudinal associations among bullying, homophobic teasing, and sexual violence perpetration among middle school students. J Interpers Violence. 2015;30:2541–61. 25315484 10.1177/0886260514553113 PMC4699677

[53] Hunehäll Berndtsson K, Odenbring Y. They don’t even think about what the Girl might think about it’: students’ views on sexting, gender inequalities and power relations in school. J Gend Stud. 2021;30:91–101.

[54] Bucchianeri MM, Eisenberg ME, Wall MM, Piran N, Neumark-Sztainer D. Multiple types of harassment: associations with emotional well-being and unhealthy behaviors in adolescents. J Adolesc Health. 2014;54:724–9. 24411820 10.1016/j.jadohealth.2013.10.205 PMC4107652

[55] Rueger SY, Malecki CK, Pyun Y, Aycock C, Coyle S. A meta-analytic review of the association between perceived social support and depression in childhood and adolescence. Psychol Bull. 2016;142:1017–67. 27504934 10.1037/bul0000058

[56] Liu Y, Gong R, Yu Y, Xu C, Yu X, Chang R, et al. Longitudinal predictors for incidence of internet gaming disorder among adolescents: the roles of time spent on gaming and depressive symptoms. J Adolesc. 2021;92:1–9. 34246122 10.1016/j.adolescence.2021.06.008

[57] Wang P, Pan R, Wu X, Zhu G, Wang Y, Tian M, et al. Reciprocal associations between shyness, depression, and internet gaming disorder among Chinese adolescents: A cross-lagged panel study. Addict Behav. 2022;129:107256. 35114630 10.1016/j.addbeh.2022.107256

[58] Kimberlin CL, Winterstein AG. Validity and reliability of measurement instruments used in research. Am J Health Syst Pharm. 2008;65:2276–84. 19020196 10.2146/ajhp070364

[59] Barros AJ, Hirakata VN. Alternatives for logistic regression in cross-sectional studies: an empirical comparison of models that directly estimate the prevalence ratio. BMC Med Res Methodol. 2003;3:21. 14567763 10.1186/1471-2288-3-21 PMC521200

