# Supplementary material for: Co-occurring cyber and in-person victimisation of bullying and sexual harassment: the associations to depressive symptoms in Swedish adolescents
Source: BMC Public Health. 2025 Feb 26;25:786. doi: 10.1186/s12889-025-21989-w (PMC11863656; doi:10.1186/s12889-025-21989-w)
Supplement: Supplementary file 1 — Supplementary Material 1 [file 12889_2025_21989_MOESM1_ESM.docx]

**Appendix 1. Victimisation scales**

**Cyber sexual harassment**

If you think about the past six months ...

How often have you experienced the following behaviours against your will online, on social media, via text messages, or while playing online games?

Someone has tried to talk to you about sex even though you didn’t want to?

Someone has asked you very personal questions even though you didn't want them to?

For example, about how your body looks or about sexual things you’ve done?

Someone has asked you to do something sexual with them even though you didn’t want to?

Someone unknown has suggested that you meet?

Someone has sent you a dick pic even though you didn’t want them to?

Someone has sent pictures/videos with nudity or sexual content (apart from dick pics) to you even though you didn’t want them to?

Someone has asked for pictures/videos of you with nudity or sexual content?

Someone has threatened you if you didn’t want to send pictures/videos of yourself with nudity or sexual content?

Someone has offered payment or other compensation in exchange for you sending pictures/videos of yourself with nudity or sexual content?

Someone has spread pictures/videos of you with nudity or sexual content?

Someone has offered payment or other compensation for you to pose naked or semi-naked and/or do something sexual in front of a webcam?

Someone has spread rumours or gossiped about your body in a sexual way, about your sexuality, or about your sexual experiences?

Someone has called you a whore/cunt/dick or similar?

Someone has called you lesbian or gay or similar?

Response alternatives: Never, Once, Sometimes, Many times

**Cyberbullying**

If you think about the past six months ...

How often have you experienced the following behaviours against your will online, on social media, via text messages, or while playing online games?

Someone has written something mean and/or hurtful to or about you.

Someone has spread rumours or gossip about you in a mean and/or hurtful way.

Someone has threatened you.

Someone has sent you pictures or videos that were mean and/or hurtful.

Someone has uploaded/spread pictures or videos that were mean and/or hurtful aimed at you.

Someone created a fake account pretending to be you to be mean and/or hurtful to you.

Response alternatives: Never, Once, Sometimes, Many times

**In-person sexual harassment**

If you think about the past six months ...

How often have you experienced the following against your will that others have:

Given sexual comments, jokes, or gestures to you?

Spread unwelcome sexual rumours about you?

Called you gay or lesbian or something similar?

Called you a whore, cunt, dick or similar words?

Spied on/filmed/taken a photo of you in a dressing room or shower?

Showed part of their body, for example, breast or genitals, to you?

Looked at you in a way that felt intrusive and sexual?

Touched‚ grabbed‚ or pinched you in a sexual way?

Pulled at/off your clothes in a sexual way (e.g., pulled at bra straps, pulled at underwear, or pulled up your skirt)?

Intentionally brushed against you in a sexual way?

Cornered you or something similar in a sexual way?

Tried to kiss or hug you?

Response alternatives: Never, Once, Sometimes, Many times

**In-person bullying**

If you think about the past six months ...

How often have you experienced the following:

Been teased or called names in a way that felt mean and/or hurtful?

Had someone/some people spread rumours or gossip about you in a way that felt mean and/or hurtful?

Been left out of a group or ignored in a way that felt mean and/or hurtful?

Been hit, kicked, pushed, scratched, cut, had your hair pulled, or shaken violently by someone/some others?

Been threatened?

Had things broken, damaged, or stolen?

Response alternatives: Never, Once, Sometimes, Many times
